# Recent and massive invasion of *Aedes* (*Stegomyia*) *albopictus* (Skuse, 1894) in Phnom Penh, Cambodia

**DOI:** 10.1186/s13071-021-04633-5

**Published:** 2021-02-18

**Authors:** P. O. Maquart, D. Fontenille, S. Boyer

**Affiliations:** 1 Medical and Veterinary Entomology Unit, Institut Pasteur du Cambodge 5, Blvd. Monivong, BP 983, Phnom Penh, 12201 Cambodia; 2 MIVEGEC, Université de Montpellier, IRD (Institut de Recherche et du Développement), CNRS, 911 Avenue Agropolis, 34394 Montpellier, France

## Abstract

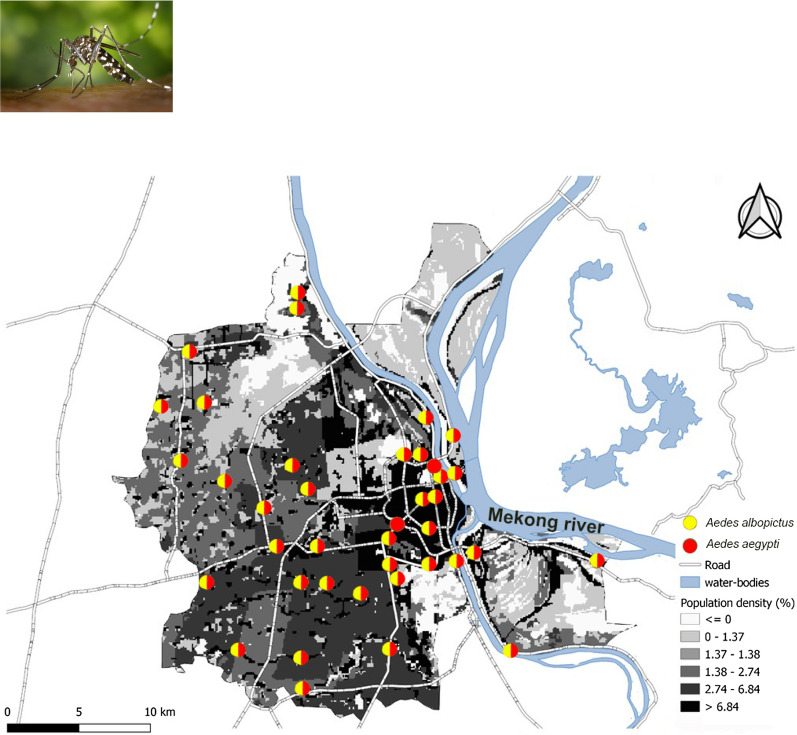

## Short note

Dengue fever and dengue haemorrhagic fever are two of the most important mosquito-borne viral diseases of public health significance [1, 2]. Their geographical spread is increasing: while only 5 countries documented dengue cases in the 1950s, more than 100 countries reported the incidence of dengue fever and dengue haemorrhagic fever in 2005 [3]. In Cambodia, since the massive epidemic in 1995, accounting for more than 400 deaths, the number of cases has been monitored every year [4, 5]. Major dengue epidemics outbreaks happened in 2007 (39,618 cases with 396 deaths), 2012 (42,362 cases with 189 deaths) and 2019 (68,597 cases with 48 deaths) (Ministry of Health, Phnom Penh, Cambodia). In 2018 and 2019, the capital Phnom Penh city was terribly affected as never before with respectively 9445 and 9298 cases (Ministry of Health, Phnom Penh, Cambodia).

Hosting about 2.13 million of the 15.3 million inhabitants of Cambodia, Phnom Penh is a rapidly developing city [6]. The multiple potential mosquito breeding sites created in the urban centre can favour vector proliferation, particularly dengue vectors [7].

The two main mosquito species responsible for the transmission of dengue virus are *Aedes aegypti* (Linnée, 1789) and *Aedes albopictus* (Skuse, 1894) [8–10]. The latter species, originating from the forests of Southeast Asia, where it was likely zoophilic (i.e. feeding on wildlife), progressively adapted to anthropogenic changes to the environment, which provided alternative blood sources (domestic animals and humans) and water collections for larval habitats [9, 11]. This species is a competent vector for all four serotypes of dengue and can transmit at least 22 arboviruses [9, 12]. Human migration favoured its spread into new areas, and it rapidly became an opportunistic container breeder, using either natural or artificial containers, having the ability to survive in small collections of water in tires, plastic buckets, and plastic cups. Today, it mainly occurs in suburban and rural areas [9].

While *Ae. albopictus* is known to originate from Southeast Asia, and known from Cambodia [13], its presence was never attested in the capital city. In 1966, its presence was declared in the rural part of Chrui Chang War, a district facing Phnom Penh, located on the eastern side of the Mekong River [14]. Despite the extensive work realised in the beginning of the 2000s [7, 13, 15, 16] in which the distribution, occurrence, and genetics of *Ae. aegypti* in the Cambodian capital were extensively studied, *Ae. albopictus* was never collected inside Phnom Penh itself (Paupy, personal communication 2020).

**Fig. 1:**
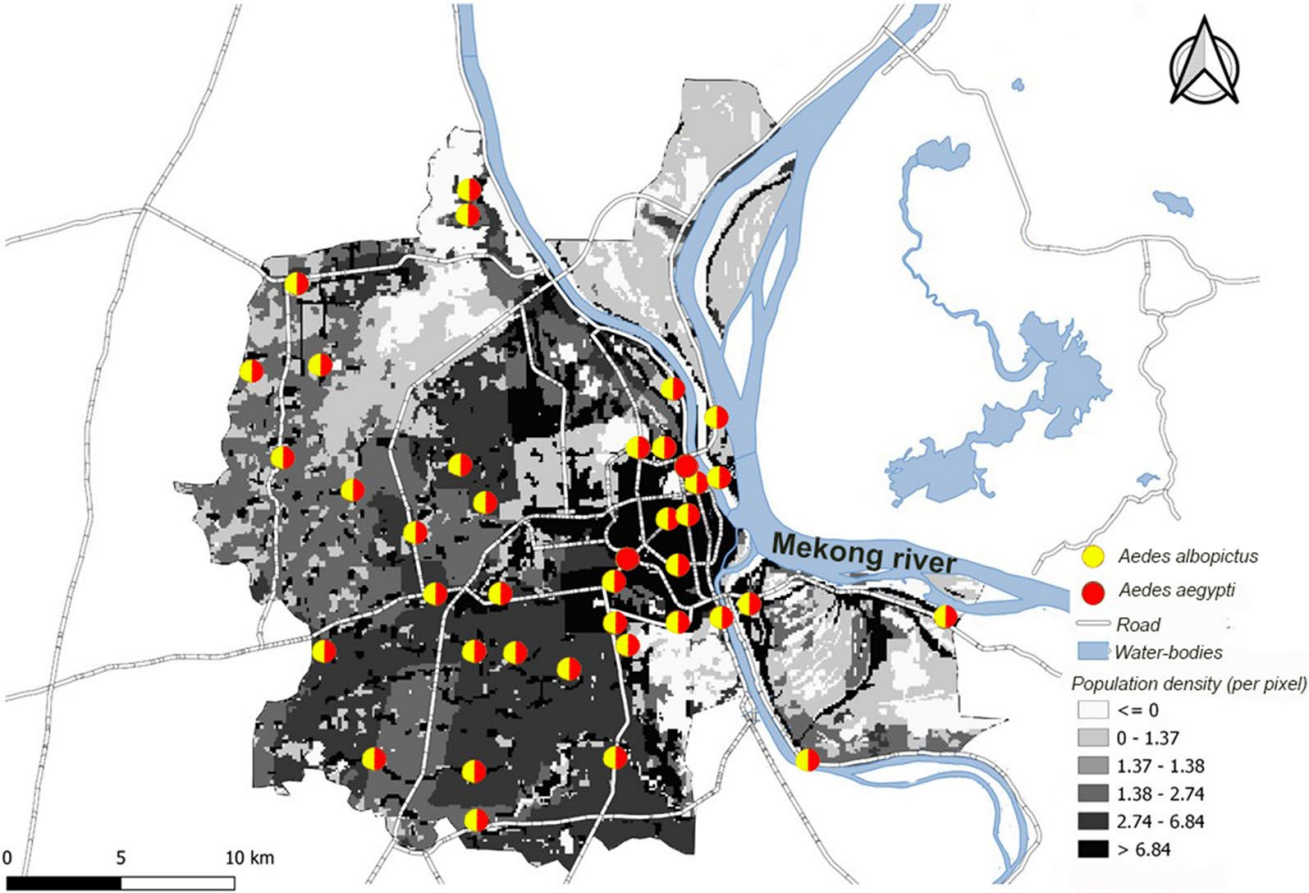
Location of the different sampling points in Phnom Penh (Cambodia) and presence of the two different species of *Aedes*: *Ae albopictus* and *Ae. aegypti.* Density data gathered from *Open Development Cambodia.* Map created via QGis.

In 2019, an entomological survey was performed by the Medical and Veterinary Entomology Unit of the Institut Pasteur du Cambodge in 42 randomly distributed sites across Phnom Penh (Fig. 1). Each location was visited every 2 months. As expected, *Ae. aegypti* was found in all sites. Surprisingly, *Ae. albopictus* was found in 40 sites including urban areas. This result attests to its recent and massive installation and distribution throughout the entire city. Meanwhile, this spreading and establishment occurred not only in Phnom Penh, but also in tropical and temperate cities worldwide [17], demonstrating that a recent invasive population has emerged [18].

Although *Ae. aegypti* and *Ae. albopictus* are competent vectors for dengue virus, it has been hypothesized that they might play different roles in contributing to a dengue outbreak. *Ae. aegypti* might initiate a cluster, leading to an outbreak, which could then be sustained by *Ae. albopictus,* expanding the scale of the virus propagation [8]. This observation made in Taiwan needs to be investigated in Phnom Penh, as does the temporal succession of each species locally.

Consequently, it is important to consider the ecology and seasonality of *Ae. albopictus* alongside that of *Ae. aegypti* when developing vector/disease control programs in Phnom Penh. This information is important for the Ministry of Health in Phnom Penh as evidence of the need to increase surveillance and control of this species in suburban and rural areas (Additional file 1: Table S1).

## Supplementary Information

**Additional file1**:** Table S1**. Total number of *Aedes aegypti* and *Aedes albopictus* collected in each trapping location inside Phnom Penh.

## Data Availability

All relevant data are within the paper and its supporting information files.
