# Supplementary material for: Recent and massive invasion of Aedes (Stegomyia) albopictus (Skuse, 1894) in Phnom Penh, Cambodia
Source: Parasit Vectors. 2021 Feb 18;14:113. doi: 10.1186/s13071-021-04633-5 (PMC7890877; doi:10.1186/s13071-021-04633-5)
Supplement: Supplementary file 1 — Additional file1: Table S1. Total number of Aedes aegypti and Aedes albopictus collected in each trapping location inside Phnom Penh. [file 13071_2021_4633_MOESM1_ESM.docx]

**Supplementary data**

Table 1 : Total number of *Aedes aegypti* and *Aedes albopictus* collected in each trapping location, inside Phnom Penh.

| Trapping location | *Aedes aegypti* | *Aedes albopictus* | **Total** |
| --- | --- | --- | --- |
| Ang Serei Suor Sdei Pagoda | 39 | 117 | **156** |
| Anhchanh Thmei Pagoda | 19 | 68 | **87** |
| Ank Mony Pagoda | 14 | 137 | **151** |
| Ank Nisai Pagoda | 13 | 45 | **58** |
| Brachum Vong Pagoda | 336 | 67 | **403** |
| Chak Ankrae Leu Pagoda | 276 | 70 | **346** |
| Champou Vorn Pagoda | 137 | 56 | **193** |
| Chas Pagoda | 145 | 9 | **154** |
| Chong Kran Ta Prom Steung Meanchey Pagoda | 273 | 0 | **273** |
| Dambok Khpuors Pagoda | 377 | 15 | **392** |
| French embassy | 253 | 138 | **391** |
| Institut Pasteur du Cambodge | 26 | 19 | 45 |
| Kampeng Pagoda | 37 | 18 | **55** |
| Koh Pagoda | 311 | 4 | **315** |
| Koh Krobey Pagoda | 147 | 40 | **187** |
| Koh Rogneang Pagoda | 51 | 17 | **68** |
| Kok Banhchon Pagoda | 67 | 10 | **77** |
| Krang Thnong Pagoda | 147 | 2 | **149** |
| Mongkol Serei Kien Khleang Pagoda | 134 | 183 | **317** |
| Mony Sar Kor Pagoda | 86 | 50 | **136** |
| Nhien Reangsei Pagoda | 131 | 1 | **132** |
| Non Mony Pagoda | 169 | 8 | **177** |
| Odoravatey Pagoda | 64 | 7 | **71** |
| Panh Jakeov Mony Russey Sanh Pagoda | 6 | 14 | **20** |
| Phnum Pagoda | 118 | 142 | **260** |
| Piphort Rangsei Pagoda | 209 | 3 | **212** |
| Praek Chrey Pagoda | 86 | 362 | **448** |
| Preah Put Khou Sacha Pagoda | 209 | 0 | **209** |
| Preah Puth Mean Bon Pagoda | 182 | 11 | **193** |
| Prey Speu Pagoda | 41 | 42 | **83** |
| Prey Veng Pagoda | 25 | 171 | **196** |
| Sak Sampov Pagoda | 27 | 243 | **270** |
| Sam Raong Kandal Pagoda | 66 | 95 | **161** |
| Sambuor Meas Pagoda | 82 | 104 | **186** |
| Snguon Pich Pagoda | 24 | 52 | **76** |
| Sovann Ampea Svay Meas Pagoda | 14 | 126 | **140** |
| Sovann Thom Reach Pagoda | 14 | 213 | **227** |
| Tain Krosang Khang Chherng Pagoda | 11 | 8 | **19** |
| Thoudoun Chamkar Dong Pagoda | 86 | 165 | **251** |
| Toul Sangke Pagoda | 283 | 2 | **285** |
| Toul Tumpong Pagoda | 500 | 5 | **505** |
| Trapeang Chhouk Pagoda | 124 | 89 | **213** |
| **Grand Total** | **5359** | **2928** | **8287** |
